# Economic Impact of COVID-19 lockdown on households

**DOI:** 10.11604/pamj.2021.40.225.27446

**Published:** 2021-12-15

**Authors:** Francis Fatoye, Tadesse Gebrye, Olujide Arije, Clara Toyin Fatoye, Omotola Onigbinde, Chidozie Emmanuel Mbada

**Affiliations:** 1 Department of Health Professions, Manchester Metropolitan University, Birley Fields Campus, Bonsall Street, Manchester, United Kingdom,; 2 Institute of Public Health, College of Health Sciences, Obafemi Awolowo University, Ile-Ife, Nigeria,; 3 Department of Medical Rehabilitation, College of Health Sciences, Obafemi Awolowo University, Ile-Ife, Nigeria

**Keywords:** COVID-19, lockdown, cost, pandemic, Nigeria

## Abstract

**Introduction:**

this study evaluated the economic impact of the COVID-19 lockdown on individuals and households.

**Methods:**

a cross-sectional online survey was used to collect data. Nigerian citizens who were domiciled or restricted from travelling abroad for no less than one month since the COVID-19 restrictions and lockdown were recruited into the study through focal persons purposively selected across the different states in Nigeria. Using WhatsApp® platform, the respondents completed the survey on household income and expenditure before and during the lockdown. Economic burden of COVID-19 lockdown on individuals and families was estimated using a prevalence-based cost of illness approach.

**Results:**

four hundred and four (male = 242; female = 162) individuals have participated in the study. The mean (SD) age of the respondents was 30.98 (10.92) years. Monthly income showed no statistically significant difference (p = 0.73) before and during lockdown. The overall household expenditure before and during the lockdown periods were USD 320 and USD 290. The total mean monthly costs for COVID-19 and non-COVID-19 health related problems were ₦11746.25 (USD30.79) and ₦11784.9 (USD 30.89), respectively. Household expenditure for hand sanitizers, facemasks, hand gloves, and disinfectants increased significantly during the COVID-19 restriction lockdown (p < 0.05). However, expenditure on education, water, electricity, fuel, internet data, clothing and wears, toiletries decreased significantly during the lockdown period (p < 0.05).

**Conclusion:**

this study suggests that the costs of continuing COVID-19 restrictions could have huge economic consequences on households and health system.

## Introduction

Coronavirus disease (COVID-19) is caused by severe acute respiratory syndrome coronavirus (SARS-CoV-2) and has been declared as a global public health emergency by the World Health Organisation (WHO) and has resulted in a deadly global pandemic [1,2]. The first patient with COVID-19 was identified in Wuhan province, China in November 2019 with severe pneumonia [3]. The symptoms of COVID-19 appear after a period of incubation of about 5 days [4]. With fever, cough and fatigue as the common symptoms [2]. Other symptoms may include headache, sputum production, diarrhoea, haemoptysis and dyspnoea [5]. There are currently over 64,615,624 confirmed cases diagnosed with the disease, and over 1,495,311 deaths are due to COVID-19, and these numbers are predicted to rise significantly in the near future [6].

Being the greatest pandemic of modern times, COVID-19 has affected more than 218 countries including low and middle income countries (LMICs) such as Nigeria. As the second-most affected country, next only to South Africa, in the sub-Sahara Africa region, Nigeria´s preparedness for the critical care of COVID-19 cases is still poor and inadequate [7]. As it currently stands, effective prevention and cure for COVID-19 is a front burner subject of research. However, approaches such as “social distancing” coughing hygiene, hand washing, wearing of masks, as well as partial or total lockdown restrictions were recommended by the WHO to reduce or interrupt COVID-19 transmission from person to person [8].

Social distancing measures and the lockdown restrictions in many countries all over the world including Nigeria have potential economic impact which has the tendency to exacerbate the already existent socio-economic burden. For example, in Nigeria lockdown has resulted to members of the public staying at home, closure of markets, workplaces, educational institutions, non-essential businesses and places of worship. In addition, other social events resulting to mass gatherings such as wedding and funeral ceremonies suspended.

It has been estimated that the economic impact of the disease globally to individuals, households, health systems and governments is between USD1-2.7 trillion [9]. Whilst lockdowns have yielded positive results in many countries in reducing number of people with COVID-19 and deaths associated with the disease, however, there are significant economic implications of lockdown policy on households. These may include increased costs of staying at home and loss of earning due to not been able to go to work. In addition, whilst some countries including LMICs have introduced some “palliative” measures to reduce the economic burden of the policy on individuals and households, however, there is still significant economic impact of lockdown on individual family.

The informal sector is a significant source of employment and family income in Nigeria. There are a number of indirect costs associated with lockdowns such as productivity losses due to loss of wages [9]. Many workers and families in LMICs such as Nigeria are already paying a large proportion of their household incomes on healthcare through out-of-pocket expenditure, therefore, the economic burden of COVID-19 related lockdown may increase the demands on families and push them to further poverty [9]. World Bank has projected that Nigeria and other LMICs would face economic crisis induced by the COVID-19 pandemic and lockdown [10]. Thus, inviting studies on empirical analysis of economic burden of COVID-19 lockdowns on individuals and their households in Nigeria. The findings of the study may influence policy on alleviating the economic burden and improving health outcomes of individuals and families during the present COVID-19 regime and other by pandemics.

## Methods

**Study site and population:** this study was a national online cross-sectional survey on the economic burden of COVID-19 lockdown on households. Respondents for this study were Nigerian citizens who were currently domiciled or restricted from travelling abroad for no less than one month since the COVID-19 restrictions and lockdown. Ethical approval for this study was obtained from the Obafemi Awolowo University, Ile-Ife Nigeria (IPHOAU/12/1553). Consent was obtained from the respondents by clicking on the online link to participate in the study. All respondents were asked to complete the questionnaire and have asked to complete their household income and expenditure before and during the lockdown.

**Eligibility criteria:** Nigerian who were 18 years and older, using a smartphone and literate in English language were eligible for participation in the study. These individuals must have been in lockdown because of COVID-19. Individuals who were in partial lockdown were excluded from the study.

**Sampling technique:** consecutive sampling technique were used to recruit respondent for the study. Contacts or focal persons across the different states in Nigeria were purposively recruited and enlisted in the study. These focal persons helped to facilitate recruitment of respondents by using their personal WhatsApp® accounts to send the survey invite/link to their personal contacts and groups that fulfilled the eligibility criteria [11].

**Sample size determination:** based on a United Nations WhatsApp Surveying Guide [11], a sample size of between 340 and 510 respondents has been recommended for a WhatsApp user population number 2000 and 3000. We improved the spread and representative of the study population by first identifying contacts persons across the nation that will power the WhatsApp® survey tool broadcasting and re-broadcasting.

**Study perspective:** a prevalence-based cost of illness approach were used to estimate the economic burden of COVID-19 lockdown on individuals and families [12]. A questionnaire, developed and validated at Manchester Metropolitan University, United Kingdom were utilised to collect information on household expenditure, including direct medical and non-medical costs as well as productivity loss associated with COVID-19 lockdown. Direct medical costs included were diagnostic test, consultation charges, hospitalisation, drug or medication, and herbal medicine. Direct non-medical costs included were transportation cost for health related problems. Indirect costs were those related to income or productivity loss and were measured by applying the human capital approach [12]. Income loss for paid workers were measured by multiplying the number of lost working hours due to COVID-19 with the actual wage rate of the respondent and members of household. The average monthly wage rate for adults older than 18 years in Nigeria was N30,000 (USD 78.6). In order to obtain the daily wage we divide for the number of working days. All costs were converted to USD using exchange rate for 2020 (USD1 = ₦381.5). The reference year for all cost estimates in this study is 2020.

**Data analysis:** data were presented as a total and as an average with a standard deviation (SD) in local currency and US dollars (USD) applying the exchange rate. Inferential statistics of t-test and ANOVA were also used to analyse the data. Data analysis was performed using the IBM SPSS 23.

**Ethical approval:** ethical approval for this study was obtained from the Health Research Ethical Committee of the Institute of Public Health, Obafemi Awolowo University, Nigeria (Registration number: IPHOAU/12/1553).

## Results

Four hundred and four (male = 242; female = 162) individuals across the different states of Nigeria have participated in the study (Table 1). The mean (SD) age of the individuals was 30.98 (10.92). The occupation of the individuals were civil servant (32%), journalists (2%), retired (5%), banker/finance worker (15%), trade (7%), self-employment/business (20%), unemployed (17%) and other (96%). Monthly income showed no statistically significant difference (p = 0.73) before and during lockdown.

**Table 1:** characteristics of the respondents

Variable			
Age (mean (SD))	30.98 (10.92)		
States (n)	36		
Income (per month)	Before lockdown Mean (SD)	During lockdown Mean (SD)	p-value
	₦263389.27 (USD690.4) (₦2589649.2)	₦207704.9 (USD544.4) (₦1846806.84)	0.73
Occupation (n (%))			
Civil servant	130 (32)		
Journalist	2 (0.5)		
Retired	5 (1.2)		
Banker/finance worker	15 (4)		
Trade	7 (1.7)		
Self-employment/business	82 (20)		
Unemployed	67 (17)		
other	96 (24)		

₦ = the currency of Nigeria, USD = United States Dollar

**Household expenditure:** the mean (SD) of household expenditure before and during lockdown is presented in Table 2. The mean values for household expenditure for hand sanitizers, toiletries, facemasks, hand gloves, and disinfectants increased significantly (p ≥ 0.05) during the COVID-19 restriction. On the other hand, the expenditure on education, water, electricity, fuel for generating set, internet data, clothing and wears, toiletries, decreased though not statistically significantly (p > 0.05). Further, expenditure on food, telephone/mobile recharge, house-rent, transportation, and entertainment increased during the COVID-19 lockdown period, though not significantly (p > 0.05). There were no statistically significant differences (p = 0.383) before and during lockdown for the overall household expenditure.

**Table 2:** household expenditure

Variable	Household expenditure		
	Before (Mean (SD)), (₦)	During (Mean (SD)), (₦)	p value
Food	42925.53 (54232.7) USD112.52 (142.16)	43454.5 (117652.31) USD113.9 (308.4)	0.945
Education	28798.1 (64609.8) USD75.5 (169.36)	16146.49 (57505.35) USD42.32 (150.7)	0.30
Water	3379.2 (5686.33) USD8.86 (14.91)	3138.9 (6302.48) USD8.23 (16.52)	0.66
Electricity	5692.4 (8864.5) USD 14.92 (23.24)	5547.51 (8359.2) USD14.54 (21.91)	0.86
Fuel for generating set	5547.51 (8359.3) USD14.54 (21.91)	5343.8 (7564.64) USD14.01 (19.83)	0.92
Internet data	7175.68 (17646.4) USD18.81 (46.26)	6604.5 (8270.5) USD17.3 (21.68)	0.67
Telephone/mobile bill recharge	3758.9 (4748.2) USD9.85 (12.45)	3806.4 (4739.53) USD 9.98 (12.42)	0.92
House rent	19255.48 (51850) USD 50.47 (135.91)	21223.1 (58441.8) USD 55.6 (153.19)	0.71
Transportation	4922.18 (9040.7) USD 12.9 (23.7)	5167.1 (9318.4) USD 13.54 (24.43)	0.78
Clothing and wears	4922.2 (9040.7) USD 12.9 (23.7)	3495.2 (6867.3) USD 9.16 (18.0)	0.08
Toiletries	3871.9 (4529.9) USD 10.15	3124.1 (3790.1) USD 8.19 (9.93)	0.05
Hand sanitizers	1538.1 (1932.4) USD 4.03 (5.07)	2075.2 (2632.5) USD 5.44 (6.90)	0.014
Face masks	1179.4 (2036.5) USD 3.09 (5.34)	1755.5 (2748.5) USD 4.6 (7.20)	0.011
Hand gloves	772.4 (2740.9) USD 2.02 (7.18)	1018.6 (1935.1) USD 2.67 (5.07)	0.021
Disinfectants	1343.8 (1771.8) USD 3.5 (4.64)	1810.7 (2395.1) USD 4.75 (6.28)	0.022
Entertainment	3278.9 (3997.9) USD 8.6 (10.48)	3581.8 (5897.4) USD 9.4 (15.46)	0.514
Total	122089.73 (141380.8) USD 320 (370.59)	110427.9 (170724.4) USD 289.46 (447.51)	**0.383**

₦ = the currency of Nigeria, USD = United States Dollar

**Direct and indirect cost of COVID-19 lockdown:** the direct medical and non-medical treatments for COVID-19 and non-COVID-19 health related problems during the lockdown were presented in Table 3. Total mean (SD) monthly costs for COVID-19 and non-COVID-19 health related problems in our study were ₦11746.25 (USD 30.79) (15390.3) and ₦11784.9 (USD 30.89) (13276.79), respectively. Except the average cost of drug/medication, the costs of diagnostic tests, consultation charges, hospitalizations, herbal medicine, and transportations were not significantly different (p ≥ 0.05). The analysis also showed that there was no statistically significantly difference (p = 0.99) between the total cost of direct and non-direct medical costs of health related problems. The average days lost due to COVID-19 lockdown were approximately 24.6 working days representing USD78.6 productivity loss.

**Table 3:** direct medical and non-medical treatments costs during lockdown for COVID-19 and non-COVID-19 health related problems

Variables	COVID-19 related health problems (Mean (SD)) (₦)	Non- COVID-19 related health problems (Mean (SD)) (₦)	p values
Cost of diagnostic tests	7863.64 (11440.2) USD 20.61 (29.99)	5385 (8702.2) USD 14.12 (22.81)	0.37
Consultation charges	3378.9 (337.23) USD 8.86 (0.88)	2882.5 (2672.4) USD 7.56 (7.0)	0.569
Cost of hospitalisation	5866.7 (5409.7) USD 15.4 (14.2)	7785.53 (6548.16) USD 20.41 (17.16)	0.41
Cost of drug/medication	3161.4 (2859.9) USD 8.29 (7.5)	5172.3 (4358.96) USD 13.6 (11.43)	0.048
Cost of herbal medicine	3161.4 (2859.9) USD 8.3 (7.5)	2541.4 (2731.89) USD 6.7 (7.2)	0.466
Cost of transportation for health related problems	2345.4 (2397.14) USD 6.15 (6.3)	2312.5 (1942.8) USD 6.06 (5.1)	0.955
Total	11746.25 (15390.3) USD 30.79 (40.34)	11784.9 (13276.79) USD 30.89 (34.8)	0.988

₦ = the currency of Nigeria, USD = United States Dollar

## Discussion

The aim of this study was to evaluate the economic impact of COVID-19 lockdown on individuals and households in Nigeria. Accordingly, a questionnaire was utilised to collect information on household expenditure, including direct medical and non-medical costs as well as productivity loss associated with COVID-19 lockdown. In an interconnected world economy, COVID-19 pandemic is a global shock to individuals, household and the society as a whole.

In line to the findings of our study, many studies have suggested that COVID-19 lockdown has led to an economic crisis to individual´s and households for food and non-food expenses. A one-week closure of schools in Taiwan [13] led to a loss of 18% of income. A four-week closure of New York City also resulted to an economic cost of $1.1bn [14]. The reduction in household expenditure for some of the food and non-food items such as education, water, electricity, clothing and wears observed in this Nigerian study may be due to self-isolation and quarantine policies in place during lockdown (Yap, 2020). We expected expenditure on education to increase during the lockdown, however, minimal home schooling was taking place due to the lack of appropriate platform for educational purposes. On the other hand, expenditure on household items such as toiletries, hand sanitizers, face masks, hand gloves, disinfectants and entertainment increased, this might be due to a relatively high level of usage of the items in order to prevent the spread of COVID-19 and increased prices during the pandemic [15], however, these were not statistically significant.

According to Haque and colleagues, there was an increased utilization and price increase for items such as antibiotics, analgesics, vitamins and personal protective equipment in Bangladesh [16]. With regard to the significant difference observed for the costs of drugs/treatment for COVID-19 health and non-health related problems in our findings, a similar results were reported in Nigeria that there was an increase in the price of medicines during lockdown [17]. An anecdotal observation in this study setting indicated that there was a panic buy of certain drugs such as those containing chloroquine and hydroxyl-chloroquine, vitamins and zinc owing to speculative reports of their efficacy to treat COVID-19. Consequently, the prices of these drugs skyrocketed.

This is the first study in LMICs for COVID-19 that has investigated the economic burden of impact of COVID-19 lockdown on individuals and households. The main limitation is that the study lasted for 4 weeks in many states, therefore, the overall and long-term economic burden of the lockdown on individuals and households might have been underestimated. The direct medical and non-medical costs of COVID-19 lockdown in this research study were estimated using the subjective opinion of the patients, and this may have been inaccurate. Further, our sample included 404 participants, limiting the generalizability of the study findings. These limitations could be addressed in future studies.

## Conclusion

COVID-19 imposes a significant burden on the households in Nigeria. Improved understanding of the economic cost of COVID-19 helps to inform and motivate policy makers to identify the best strategies to alleviate the economic burden associated public health crisis. Given the high economic impact of the pandemic to individuals and most people in LMIC countries work in informal sector, policy makers and governments are to put in place strategies that will help these individuals and households through providing financial support to them.

### What is known about this topic

- COVID-19 a new kind of coronavirus was declared a pandemic by the World Health Organization, and has shown exponential growth in Africa.

### What this study adds

- The continuing COVID-19 restriction could have huge economic consequences on the individuals and households.
